# Real-time full-color meta fiber endoscopy

**DOI:** 10.1038/s41377-023-01222-2

**Published:** 2023-07-24

**Authors:** Changyuan Yu, Zhi Cheng, Jiaqi Qu

**Affiliations:** Photonics Research Institute, Department of Electrical and Electronic Engineering, The Hong Kong Polytechnic University, Hong Kong, China

**Keywords:** Imaging and sensing, Interference microscopy

## Abstract

The remarkable capacity of metasurfaces to exert precise control over the propagation of light has ushered in a realm of unprecedented possibilities for “Lab-on-fiber”, and in this work, showcased real-time full-color imaging in a meta-optical fiber endoscope.

## Abstract (web summary)

A real-time full-color fiber endoscopy is realized by an inverse-designed metalens combined with a coherent fiber bundle.

Metasurfaces represent a distinctive category of quasi-two-dimensional devices characterized by artificial subwavelength micro-nanostructures that afford them the remarkable capability to flexibly manipulate electromagnetic waves, which has garnered extensive scientific attention over the past decade, leading to the practical realization of numerous photonic devices. In contrast, optical fibers have enjoyed over half a century of development in both industrial and research communities, primarily serving as a medium for light transmission. The convergence of metasurfaces and optical fibers opens the door to new research area with enhanced functionality and transmission. Recently^1^, Arka Majumdar and collaborators demonstrated a real-time full-color meta-optical fiber endoscope developed by inverse-design engineering which enables a 33% reduction in the rigid tip length compared with the traditional gradient-index (GRIN) fiber endoscope as illustrated in Fig. 1. Such downscaling is crucial for the practical applications of fiber endoscopes.

**Fig. 1:**
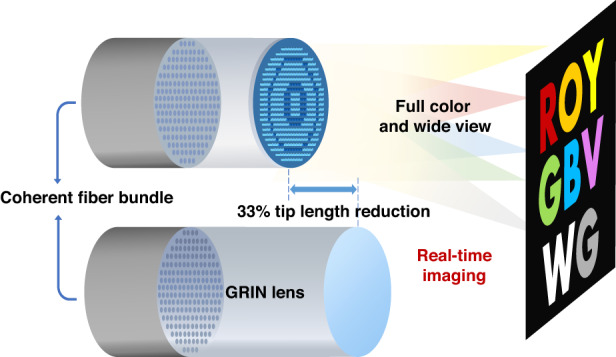
The proposed meta-optical fiber endoscope. An inverse-designed meta-optic is combined with a coherent fiber bundle for real-time imaging. Compared to a conventional GRIN lens, 33% tip length reduction is achieved while maintaining comparable imaging performance

One of the biggest merits of meta-optics is the ability to compress the thickness of an optical system, and in this study, the rigid length of a fiber endoscope is greatly decreased with the assistance of a metalens. However, in the design of an achromatic metalens, there is a limitation between the bandwidth and the numerical aperture (NA)^2^. Generally, it is almost impossible to obtain a full-color metalens with the NA large enough for an endoscope in the visible light region. To overcome this obstacle, this inverse-design methodology utilized in this work optimizes the modulation transfer function (MTF) as the objective function instead of solely considering chromatic aberrations. The phase profile is obtained by optimizing the figure of merit (FOM) using a gradient descent algorithm, and appropriate unit cells matching the modified phase profile obtained from a pre-trained neural network are picked for the next iteration. The optimization process strikes a delicate balance among various factors within the imaging system, encompassing working distance, resolution, aberrations, and more, to achieve a satisfactory imaging quality specifically tailored for endoscopic applications. In other words, the imaging quality at different incident angles is improved by sacrificing chromatic aberration correction to some extent. With the help of inverse design, such a full-color metalens with a diameter of up to 1 mm can be designed regardless of the sampling constraints^3^. However, a support platform to set up this 1-mm-diameter metalens is required because the core diameter of a single fiber is typically micro-scale, e.g., 9 μm in a single mode fiber and ~50 μm in a photonic crystal fiber. To do this, this team enables the coherent fiber bundle as the meta-optics substrate platform as well as the signal transmittance medium, which could also collect more information than a single fiber in the application of endoscopy. One can get a quick overview of the research of fiber-meta tips (FMT) by the ref. ^4^, and the primary obstacle lies in the fabrication process, stemming from the fragility of optical fibers and the limited fabrication area of fiber facets in comparison to silicon wafers used in CMOS processing. Various direct and indirect fabrication methods have been proposed, including top-down, bottom-up, and indirect transfer methods. Fiber bundles could be another good choice for FMT fabrication where meta-optics in macroscopic-scale is easier to align and attach to the end of optical fibers. The combination of metalens and coherent fiber bundle is nearly perfect for solving several problems simultaneously in this work.

The proposed research can be evaluated by comparing it with a previous study conducted by Federico Capasso and Melissa J. Suter’s research groups^5^, which is also excellent research on the FMT endoscope. Both works leverage the inherent capabilities of optical fibers in signal transmission and exploit the imaging potential of meta-optics. The working bandwidth and chromatic dispersion are relatively ignored in the comparing work. Conversely, the integration of optical coherence tomography (OCT) offers a more comprehensive information set for medical practitioners. However, like other computational imaging techniques, OCT is non-real-time due to the time cost involved in the process of image reconstruction. In contrast, this work with meta-optical fiber presents a true real-time imaging process.

While both endeavors are exceptional and captivating, it is important to acknowledge that they represent the initial stages rather than a conclusive endpoint. As an ideal solution for endoscopy, FMT can be further enhanced through the development of more fabrication-friendly methods and practical implementations. A prospective notion entails the integration of Raman spectroscopy onto the facet of optical fibers or fiber bundles, thereby enabling real-time histologic tissue diagnosis. Taking a broader outlook, fiber-meta tips possess a significant untapped potential for advancement in optical communications and sensing applications.

The advent of FMT has engendered significant interest, as their potential to augment the combined effect of fibers and metasurfaces surpasses the mere summation of their individual contributions. This study unveils a transformative approach wherein the rigid tip length of an endoscopic device is markedly reduced through the integration of a coherent fiber bundle and meta-optics. This breakthrough innovation paves the way for the realization of diverse forms of FMT, thereby facilitating a plethora of functionalities spanning imaging, optical communication, and sensing applications.
